# What Keeps Kids Coming Back? Retention in a Sport-Based Positive Youth Development Program

**DOI:** 10.3389/fspor.2022.816539

**Published:** 2022-07-20

**Authors:** Dawn Anderson-Butcher, Anthony J. Amorose, Claire Sobecki, Travis R. Scheadler, Obidiah Atkinson, Emily Gutzwiller

**Affiliations:** ^1^College of Social Work, The Ohio State University, Columbus, OH, United States; ^2^School of Kinesiology and Recreation, College of Applied Science and Technology, Illinois State University, Normal, IL, United States; ^3^Department of Human Sciences, College of Education and Human Ecology, The Ohio State University, Columbus, OH, United States

**Keywords:** retention, disadvantaged populations, youth sport, positive youth development, life skills

## Abstract

Research finds engagement in sport-based positive youth development (PYD) programs contribute to key outcomes related to physical, social, emotional, cognitive, and mental health. Consistent, long-term participation ensures youth, especially those who are socially vulnerable, reap the most benefits. Even when common barriers are removed, retention remains a challenge. Using mixed methods, this study explored factors related to long-term retention among youth from socially vulnerable circumstances attending one sport-based PYD program. Factors related to youth participation in the previous year's program, as well as general youth demographics, were examined using difference tests and binomial logistic regression to explore retention among 124 of the 384 youth who returned to the program the following year. Results of the regression analyses showed the full model (with all predictors included), vs. an intercept-only model, was statistically significant, χ^2^ (11, *N* = 235) = 23.38, *p* = 0.02. The model correctly classified 88.2% of the non-returners and 28.0% of the returners for an overall correct classification rate of 67.2%. Better fitness levels, higher perceived social responsibility (an outcome targeted in the program), and some demographic variables (such as lower poverty rates and younger age) were associated with a greater probability of returning, although effect sizes were small. Additionally, interviews were conducted with 18 parent/caregivers of returning youth and 18 match comparison parent/caregivers of non-returning youth. Qualitative analyses revealed few differences in previous year's program experiences between returners and non-returners, as well as similarities in reported benefits from involvement. Both sets of parent/caregivers cited positive experiences overall, and particular benefits related to meeting new people and learning new sports. Parents/caregivers of non-returners, however, noted the value of physical literacy components of the program more so than their counterparts. Social interactions, both positive and negative, seem to have particular relevance for retention. Findings overall, however, demonstrate challenges with predicting retention and fostering long-term engagement among youth from socially vulnerable circumstances in programming.

## Introduction

Youth from low-income communities often lack access to resources and opportunities to engage in physical activity and sport, thereby limiting growth in physical literacy, physical and mental health, and other positive developmental outcomes (Eime et al., 2013; Bantham et al., 2021; Kuhn et al., 2021). Although some research suggests participation in sport may contribute to negative outcomes such as increased anxiety, negative affect and problem behaviors (Brustad et al., 2001; Eccles et al., 2003), sport when designed with quality and intentionality is one social setting fostering positive developmental outcomes (Coalter, 2012; Bean and Forneris, 2017; Anderson-Butcher, 2019). Many young people, however, experience barriers limiting their involvement in sport, ones related to cost, transportation, lack of safe spaces to play, and limited availability of programming (Bantham et al., 2021).

Sport-based positive youth development (PYD) programs are designed to address these barriers and increase access among socially vulnerable youth (Eime et al., 2013; Anderson-Butcher, 2019; Whitley et al., 2019). These programs incorporate best practices in PYD, ones such as fostering initiative, relationships, and belonging, as well as addressing risks and promoting protective factors (Eccles and Gootman, 2002; Catalano et al., 2004; Lerner et al., 2005). Sport-based PYD programs also draw upon sport's innate features by prioritizing teamwork, discipline, communication, and goal-directed behaviors (Anderson-Butcher, 2019; Whitley et al., 2019). One of the most common sport-based PYD programs is Hellison's s Teaching Personal Responsibility through Sport model (TPSR, Hellison, 2011). TPSR is widely used across the world and one of the most researched frameworks demonstrating impact (Pozo et al., 2018).

Increasing evidence supports the role of TPSR and other sport-based PYD in promoting fundamental motor skills, physical literacy, physical competence, physical self-worth, physical fitness, social-emotional learning skills, and prosocial behaviors, especially among socially vulnerable youth (e.g., Eime et al., 2013; Anderson-Butcher et al., 2014a, 2018, 2020; Hermens et al., 2017; Whitley et al., 2019; Lower-Hoppe et al., 2020; Warner et al., 2021).

Fostering long-term participation in sport-based PYD programming, as well as sport, physical activity, and PYD programs in general, continues to be a challenge (Anderson-Butcher, 2005; Witt and Dangi, 2018). In fact, there is limited longitudinal research exploring outcomes and processes associated with involvement in sport and sport-based PYD, in general (Anderson-Butcher, 2019; Whitley et al., 2019). Research does point out, however, that there must be sufficient dosage to produce desired outcomes (Catalano et al., 2004; Anderson-Butcher, 2019). Long-term engagement, especially among youth from socially vulnerable circumstances, remains a challenge (Anderson-Butcher et al., 2018). Therefore, understanding the factors that influence long-term participation among hard-to-serve groups is an increasing priority (Anderson-Butcher et al., 2020). As such, this study begins to explore factors related to long-term retention among youth from socially vulnerable circumstances attending one urban sport-based PYD program located in the Midwest. We explore personal and structural predictors of retention through quantitative analyses, as well as qualitatively distill forces and factors perceived by parents/caregivers influencing long-term engagement.

### The Challenge of Dropout

The value of sport-based PYD programs, especially for those serving youth from socially vulnerable circumstances, is increasingly clear. To successfully achieve desired outcomes, however, youth must engage in consistent and regular opportunities over-time (see Bean and Forneris, 2017; Pierce et al., 2017, 2019; Anderson-Butcher, 2019). Less-than-perfect participation and retention rates challenge the ability of sport-based PYD programs to promote desired outcomes even when these programs reduce many known barriers to participate. Indeed, dropout has long been a concern in youth programming and research, given “youth will not benefit from these programs if they are not there” (Anderson-Butcher, 2005, p. 6).

Research in the broader field of PYD points to the value of dosage and long-term involvement. For instance, Hansen and Larson (2007) studied 1,822 high schoolers and found that the amount of time spent in organized activities (including sports) related to self-reported positive outcomes such as academic achievement and self-esteem. Dosage also was related to enjoyment, motivation, and the demonstration of leadership, suggesting more meaningful engagement occurred with increased participation. Relatedly, Marsh and Kliettman (2002) found total time in extracurricular activities over multiple years was significantly related to positive changes on multiple youth development outcomes, including academic performance, self-esteem, substance use, college/career readiness.

Further, PYD research further demonstrates the value of retention. Specifically, Grossman and Rhodes' (2002) rigorous longitudinal study on mentoring in the Big Brother Big Sister Association demonstrate how outcomes are progressively greater as relationships persist for longer periods of time (specifically, a year or longer). In contrast, youth in relationships <3 months showed declines in self-esteem and other PYD outcomes as compared to the control group. Interesting youth in the study coming from socially vulnerable backgrounds were more likely to have pairs discontinue, demonstrating challenges again for serving underserved populations (Grossman and Rhodes, 2002). Others have confirmed these findings suggesting longer-term mentoring relationships result in the better outcomes, as well as identified issues with earlier termination among youth from diverse backgrounds (DuBois and Rhodes, 2006; Grossman et al., 2012).

Some suggest the concepts of dosage and time are essential to foster long-term relationships, a sense of belonging, promote bonding and the adoption of prosocial norms, and develop competence through learning and application (Hawkins et al., 1992; Larson and Verma, 1999; Catalano et al., 2004). Catalano et al. (2004), in fact, reviewed the PYD literature finding well-effective school-based PYD programs were delivered over a period of 9 months or longer, whereas non-school programs averaged about 12 sessions per intervention in dosage.

Although not as common, there has been some research in youth sport, specifically, documenting the costs associated with dropout. Specifically, Vella and colleagues have conducted two different longitudinal studies and found dropout contributes to broader long-term negative outcomes among youth, such as reduced physical activity, greater body fat, and increased mental health symptomology later in life (Vella et al., 2014, 2015). Regardless of the studies, the key is to recruit youth to programs and promote their long-term participation and ultimate retention (Anderson-Butcher, 2005). Better outcomes occur when we do so.

### Factors Related to Retention

So what prevents long-term involvement in sports-based PYD programs? Evidence suggests participation and retention vary based on different background and characteristics of youth. Foremost is income, as youth living in poverty tend to have less opportunities for prosocial engagement, less resources to support involvement, and more exposure to risks that deter their involvement than their wealthier peers (e.g., Eccles et al., 2003; Lauer et al., 2006; Center for Law and Social Policy, 2015; Perks, 2020). Other barriers to retention in sport-based PYD exist such as the lack of necessary transportation, clothing, equipment, as well as physiological barriers such as higher BMIs (Ullrich-French and McDonough, 2013). Additionally, youth of color are disproportionately disadvantaged due to heightened exposures to poverty and anti-social behaviors, and decreased access to resources and opportunities that could reverse these outcomes (Brown and Evans, 2002; Li et al., 2007; Jain and Cohen, 2013). Further, many of the barriers also intersect. Clark et al. (2019), for example, discussed how financial support is beneficial for low-income families but does not offset other barriers that are often ignored such as enrollment fees, time constraints, and transportation demands.

Overall experiences in the sport-based PYD program over-time also may impact participation and retention. Youths' discontinuation may be related to feelings of alienation, experiences of bullying by peers at programs, conflicts with peers, and poor team dynamics (Dworkin, 2007; Fredricks et al., 2010; Crane and Temple, 2015). Positive experiences, though, may predict ongoing participation. For instance, positive youth interactions, enjoyment, and belonging are related to program attendance and continued involvement (Weisman and Gottfredson, 2001; Anderson-Butcher and Fink, 2005; Fredricks et al., 2010; Crane and Temple, 2015).

Although many strategies to promote effectiveness and retention have been identified (see Anderson-Butcher, 2005, 2019), little is known why some youth do not return. Yet the impact of sport-based PYD programs on physical literacy and other related outcomes may be limited if youth fail to return to programs in subsequent years. Better understanding retention among youth from socially vulnerable circumstances is of special importance, too, given these young people's increased exposure to adversities and risks that deter their health and well-being (Anderson-Butcher et al., 2020).

To improve our understanding of retention, the present study utilized a mixed-methods design to explore participation and retention in one sport-based PYD program. We were interested in examining multiple program and participant related factors which might impact retention. Using quantitative methods, we first explored if there were differences in backgrounds between returners and non-returners, specifically exploring if there were differences in race, poverty status, age, BMI, and pre-camp perceptions of social and sport skills. Second, we examined if returners and non-returners differed in their prior program experiences. Last, we explored if attendance, growth in social and sport skills during the program, a sense of belonging, and overall satisfaction differed among returners and non-returners. Then using qualitative methods, we interviewed parents/caregivers of returners and non-returners to explore if there were any differences in their child's program experiences to distill further insights related to retention and dropout. All study procedures were approved by the lead author's University Institutional Review Board.

## Methods

### Context

This study explored retention of youth in one sport-based PYD program called LiFE*sports*. LiFE*sports*' mission is to “prepare youth for life and leadership through sport.” Each year the program serves ~600 youth aged 9–15 from socially vulnerable circumstances each year in its 19-day summer camp. In a typical year, about 80% of youth live at or below the poverty line, 85% self-identify as Black, and about 20% of children exhibit some type of behavioral issue. LiFE*sports* is offered for free, and participants also receive free transportation to and from camp, free health physicals and dental screenings, and two meals per day during each day of programming. Essential elements of PYD programs such as intentional strategies to promote belonging, foster relationships, and build life skills are incorporated in this program designed to teach sport and social skills to underserved youth (www.lifesports.osu.edu).

At LiFE*sports*, youth participate in 15 h of a play-based social competence curriculum (called Chalk Talk) designed to enhance four specific social skills: (1) self-control, (2) effort, (3) teamwork, and (4) social responsibility (together referred to as S.E.T.S.). As an example, one of the self-control lessons focuses on recognizing emotions. Youth learn about different emotions and then share out various feelings based on different experiences (i.e., weren't invited to a party, got an A on a test). Groups then prepare and act out short role plays which involve pre-determined places and certain emotions (i.e., at the swimming pool with excitement, disappointment, or guilt). Opportunities for reflection through discussion and journaling happen throughout the session, and debriefing to support processing and the transfer of skills “outside of camp” occurs.

They also receive 5 h of instruction in 8 different sports to encourage sampling and introduce new sports youth may have not had exposure to before (e.g., lacrosse, soccer, tennis, basketball, swimming, football, volleyball, etc.). Instruction is focused on basic techniques and tactics. For instance, youth learn cues specific to sport-specific techniques (i.e., dribble the basketball with your fingertips, pass the soccer ball with your instep, etc.), as well as tactics related to offensive and defensive strategy.

Throughout the program youth also receive reinforcement and encouragement for their demonstration of S.E.T.S. As in evidence-based social skills and behavioral support interventions, verbal praise and tokens in the form of S.E.T.S. buttons aim to teach, reinforce, and support use of and growth in S.E.T.S. over the course of their participation economy. During the last few sessions, youth participate in a culminating event, the LiFE*sports* Games, where they compete in sports and Chalk Talk Challenges and are honored for their hard work and involvement. An example Chalk Talk lesson plan is provided in the Appendix.

### Quantitative Study Component

#### Procedures and Sample

To recruit youth for the study, parents/caregivers of youth participating in 2018 were asked to provide consent for their child's participation, and youth 14 and older provided assent. Participation was voluntary and did not impact youth registration. This study focused on youth who attended the LiFE*sports* summer camp for the first time in 2018, as we wanted to ensure there would be similarity in the extent of camp exposure overtime between those who returned and did not in 2019. There were 595 youth who attended the LiFE*sports* camp in 2018 with permission to participate in the quantitative research. Of those, 384 were 1st year campers who had not aged out of the program and who returned in 2019. The final sample for the quantitative component of the study included these 384 youth. The mean age of campers was 11.05 years (*SD* = 1.57). The majority were boys (59.4%), self-identified as Black (82.6%), and 40.4% reported living at or below the poverty line. A total of 124 (32.3%) of these youth returned to camp in 2019. Additional descriptive information on the final sample is reported in the result section. Youth completed surveys on the 1st day and last day of Chalk Talk. The surveys took ~45 min to complete.

#### Measures

The variables of interest in the quantitative part of the study were assessed using a variety of measurement scales and single item questions collected either in the camp registration material or the beginning and/or end of the 2018 camp. The details of the assessments are described below. Demographic information was collected, as well as self-report data on various outcomes targeted in the LiFE*sports* curriculum (i.e., social and athletic competence, belonging, etc.).

##### Demographic Information

Basic demographic information during program enrollment and included individual items asking about the camper's age, gender, and race. During registration campers' parents/caregivers reported on family income and number of people living in their household. This information was combined to determine if the camper was living at or below the poverty line using the 2018 U.S. Department of Health and Human Services Guidelines (https://aspe.hhs.gov/2018-poverty-guidelines). Height and weight were assessed during camp and used to calculate Body Mass Index (BMI) using the Centers for Disease Control and Prevention calculator (https://www.cdc.gov/healthyweight/bmi/calculator.html).

##### Self-Control

The Perceived Self-Control Scale (Anderson-Butcher et al., 2016a) was used to assess the degree to which youth perceive their ability to control and manage their reactions and emotions. Campers responded to each of the eight scale items using a 5-point Likert response format (*1* = *Not at all true* to *5* = *Really true*). An example item is “I am able to control my temper.” Within sport-based PYD programs the scale has demonstrated high factor loadings and strong internal consistency, suggesting scores on this scale are valid and reliable (Anderson-Butcher et al., 2016a). Internal consistency estimates for the present study showed acceptable levels of reliability (Cronbach's α at pre-camp = 0.88 and post-camp = 0.92).

##### Effort

The Perceived Effort Scale (Anderson-Butcher et al., 2016b) was used to measure the degree to which youth perceive their ability to exert effort and try hard across situations at the beginning and end of camp. The scale includes seven items measured on a 5-point Likert scale (*1* = *Not at all true* to *5* = *Really true*). An example item is “I try hard even when nobody (or an adult) tells me to.” Past research has shown the scores on the scale to be reliable and valid (Anderson-Butcher et al., 2016b). Internal consistency estimates for the present study show acceptable levels of reliability (Cronbach's α at pre-camp = 0.83 and post-camp = 0.89).

##### Teamwork

The Teamwork Scale for Youth (Lower et al., 2017) was used at the beginning and end of camp to assess the degree to which youth perceive their ability to collaborate and work with others to achieve a common goal in the group or team context. The scale includes eight items measured on 5-point Likert scale (*1* = *Not at all true* to *5* = *Really true*). An example item is “I treat my group members as equal members of the team.” Lower et al. (2017) have demonstrated acceptable factorial validity, measurement invariance across time, concurrent and predictive validity, as well as strong reliability for the scale within sport-based PYD programs. The scale scores in the present study demonstrated acceptable levels of reliability (Cronbach's α at pre-camp = 0.81 and post-camp = 0.88).

##### Social Responsibility

The Perceived Social Responsibility Scale (Anderson-Butcher et al., 2016c) was used at the beginning and end of the camp to measure the degree to which youth perceive they respect the rights and feelings of others. The scale includes seven items measured on a 5-point Likert scale (*1* = *Not at all true* to *5* = *Really true*). An example item is “I am concerned about others in my community.” Past research has shown the scores on the scale to be reliable and valid (Anderson-Butcher et al., 2016c). Internal consistency estimates for the present study also show acceptable levels of reliability (Cronbach's α at pre-camp = 0.84 and post-camp = 0.88).

##### Social Competence

Perceived social competence at the beginning and end of camp was measured using the 5-item Perceived Social Competence Scale-II (PCSC-II; Anderson-Butcher et al., 2014a). One example item was “I ask others if I can be of help.” Anderson-Butcher et al. (2014a) have provided acceptable factorial validity, factorial invariance across time, and strong predictive validity for the scale scores within the context of sport-based PYD programs. Internal consistency estimates for the present study also show acceptable levels of reliability (Cronbach's α at pre-camp = 0.89 and post-camp = 0.89).

##### Sport Competence

Perceived sport competence was assessed using an adapted version of the Amorose (2002). Perceived Athletic Competence Scale. Youth were asked at the beginning and end of camp how good they thought they were at the sport and physical activities included at the program (e.g., volleyball, football, swimming, social dance/hip, soccer, lacrosse, softball, basketball, running, fitness, and recreational games). Response options were based on a 5-point Likert scale (*1* = *Not good at all* to *5* = *Very good*). The scores for each individual sport/activity were averaged to reflect overall perceptions of sport competence. Internal consistency estimates for the present study revealed acceptable levels of reliability (Cronbach's α at pre-camp = 0.77 and post-camp = 0.77). This adapted version has been tested in other sport-based PYD research and shown to have adequate psychometric properties in other sport-based PYD research (Anderson-Butcher et al., 2013; Lower-Hoppe et al., 2020).

##### Belonging

A sense of belonging was measured using the 5-item Belonging Scale developed by Anderson-Butcher and Conroy (2002) at the end of camp. Example items include “I feel comfortable with people at LiFE*sports*” and “I am part of LiFE*sports*.” The measure employs a 5-point Likert scale (*1* = *Not true at all* to *5* = *Really true*). Scores on this measure have been shown to have adequate psychometric properties (Anderson-Butcher and Conroy, 2002). The internal consistency reliability was α = 0.91 in this study.

##### Attendance

Attendance at the program was taken daily. To capture each youth's level of participation, we calculated a percentage of the 19 possible days they attended.

#### Quantitative Data Analyses

Standard procedures were used for screening data prior to the main analyses (Tabacknick and Fidell, 2013). Twelve cases (6 returners, 6 non-returners) were identified as multivariate outliers (Mahalanobis Distance < 0.001) and were removed from further analyses. List-wise deletion of cases was used to deal with any missing data, which occurred inconsistently across the variables included in the study. The main analyses focused on understanding returning status. First, univariate statistics were used to look at differences between returners and non-returners. The chi-square statistic was used for looking at differences in categorical variables (e.g., gender) and *t*-tests were used for continuous variables (e.g., age). Next, binomial logistic regression analyses were used to explore whether returning status could be predicted from a set of characteristics the youth possessed entering camp and then a set of predictors based on their participation and experience during the 2018 camp. Chi-square model fit relative to a null model was examined to see if each set of predictors significantly predict the probability that a youth would return. Regression coefficients, Wald test, and odds ratios were used to examine the contribution of the specific predictor variables. Multicollinearity within the predictor variables was a non-issue with tolerance values all > 0.20 (Tabacknick and Fidell, 2013). Significance level for each analysis was set at *p* < 0.05.

### Qualitative Study Component

#### Procedures and Sample

For the qualitative component of the study, we were interested in understanding the broader familial and contextual factors influencing whether a child returns or not. As such, parents/caregivers of all 2018 camp participants who did not re-enroll in the summer of 2019 were recruited (*n* = 366). Over a 6-month period, non-returners with contact information (*n* = 345) were sent two emails inviting them to participate in an interview exploring factors associated with retention. In the case of no response, a trained research assistant conducted a follow-up recruitment phone call. After two email attempts and one phone call, a total of 18 parents/caregivers of non-returners enrolled in the study. Please note one parent/caregiver had multiple children who did not return. In this case the child born in the earliest month of the year was selected as the child to refer to in answering interview questions.

Match comparisons for the children of these 18 parents/caregivers of non-returners enrolled in the study were selected from the 2018 registration files based on age and gender. Parents/caregivers of potential matches were recruited via phone asking them to participate. If declined or unavailable, another match was selected based on the next potential participant in alphabetical order after the original match. Interviews were conducted with the 18 parents/caregivers of non-returners (mean age of the children = 10.77 years; 11 children were male; 16 self-identified as Black, 1 as Other) and 18 parents/caregivers of the match comparison children who returned (mean age of the children = 10.77 years; 11 were male; 17 Black, 1 Other).

#### Interviews

A semi-structured interview guide was used to explore parent/caregiver perceptions of their child's overall camp experience the previous summer Example interview questions include “Why did you decide to have your child return (not return) to the program?” “What did you child get form attending the program last year? What were the best and worse parts?” and “What were challenges of returning or facilitators of coming back?” Additionally, parents/caregivers of non-returners were asked specifically one additional question about whether there were any other external circumstances that prevented them from enrolling their child in the program the following year. Probes were used to gain greater insight into the parents/caregivers' perceptions of their experiences by asking for specific examples. After receiving consent, a total of 26 phone interviews were conducted, lasting ~30 min each. Specifically, interviews were not recorded but were transcribed verbatim on an interview guide in real time.

#### Qualitative Data Analysis

Non-returner transcriptions were analyzed together and the returner transcriptions separately, to identify themes and subthemes. Specifically, thematic analyses was used give its flexibility, theoretical freedom, and utility for providing rich, detailed yet complex accounts of data (Braum and Clarke, 2006). Two individuals participated in the coding process using both deductive and inductive analyses (Patton, 2015). Deductively, we coded raw data into pre-established categories (i.e., positive or negative experiences). We then utilized inductive analyses to identify themes from the data to create sets of integrated concepts and subthemes under each broader theme of positive or negative experiences. The main categorizing strategy for creating broader themes was data coding (as recommended by Maxwell, 2005). As part of the coding process, the researchers pulled direct quotes that represented each category and organized codes into lower-order sub-themes and higher-order themes (Maxwell, 2005; Creswell and Poth, 2018). Coding focused on identifying, analyzing, and reporting patterns of data, specifically looking for themes or pattern of responses through specific thematic analysis phases or steps outlined by Braum and Clarke (2006).

After the initial independent coding, the two researchers compared their coding processes and discussed discrepancies. In the few cases when discrepancies were found, the two discussed the themes and came to a mutual agreement on the final coding. As recommended by Patton (2015), researchers often use analyst triangulation methods to validate themes. In the current study, the researchers used peer and expert review to decrease bias and ensure validity (Lincoln and Guba, 1985). A senior youth engagement strategist with a local Boys & Girls Club served as a peer reviewer. Themes were discussed and confirmed during this process allowing for further validation of the accuracy of our interpretations. As recommended by Barker and Pistrang (2005), an expert reviewer with over 25 years in sport-based PYD was consulted regularly to provide feedback on the interpretation of these data.

## Results

### Quantitative Results

Descriptive statistics for the study variables are presented in Table 1 for the total sample and by returning status. In terms of characteristics entering camp, significant univariate χ^2^ differences were found for race and poverty status. Specifically, non-returners were less likely to self-report being Black relative to all other race options (which included the categories of multi-racial, Caucasian, or other) and were more likely to live at or below the poverty level than returners. Univariate *t*-tests also indicated significant difference in age, BMI, and pre-camp perceptions of sport competence. Non-returners were more likely to be slightly older and have a higher BMI while also being less likely to believe they were competent at sports. The effect sizes of these difference, nonetheless, were small with eta-squares values of 0.01, 0.04, and 0.01, respectively. The only difference in the set of participation and experience during 2018 camp variables was attendance percentage. The *t*-test indicated that returners attended a higher percentage of days during the 2018 camp relative to non-returners. Again, the effect size of this difference was relatively small (eta-squared = 0.05). All other comparisons were non-significant.

**Table 1:** Profile of campers overall and by returning status.

	**Total Sample** **(*N* = 372)**		**Non-Returners** **(*n* = 254)**		**Returners** **(*n* = 118)**		**Difference**
	***%*** **or M (SD)**	***N***	***%*** **or M (SD)**	***n***	***%*** **or M (SD)**	***n***	***p =***
**Characteristics entering camp**							
Gender							0.85^a^
Male	58.6%	218	58.3%	148	59.3%	70	
Female	41.4%	154	41.7%	106	40.7%	48	
Race							0.04^a^
Black	83.0%	307	80.2%	203	88.9%	104	
Other	17.0%	63	19.8%	50	11.1%	13	
Poverty status							0.00^a^
Living at or below poverty line	40.4%	129	47.1%	104	25.5%	25	
Living above poverty line	59.6%	190	52.9%	117	74.5%	73	
Age	11.09 (1.57)	372	11.20 (1.64)	254	10.86 (1.40)	118	0.05
BMI	21.12 (5.03)	330	21.81 (5.17)	220	19.74 (4.46)	110	0.00
Sport competence (pre-camp)	3.35 (0.69)	307	3.29 (0.71)	199	3.46 (0.65)	108	0.04
Social competence (pre-camp)	4.02 (0.88)	308	4.04 (0.89)	200	3.99 (0.87)	108	0.64
Self-control (pre-camp)	3.59 (0.92)	311	3.63 (0.89)	202	3.53 (0.98)	109	0.34
Effort (pre-camp)	4.06 (0.75)	310	4.05 (0.75)	201	4.05 (0.75)	109	0.99
Teamwork (pre-camp)	3.95 (0.74)	309	3.93 (0.76)	200	3.98 (0.71)	109	0.62
Social responsibility (pre-camp)	3.89 (0.85)	309	3.87 (0.87)	200	3.94 (0.83)	109	0.46
**Participation and experiences during 2018 camp**							
Attendance (percent of days present)	76.17 (23.47)	372	72.63 (25.74)	254	83.81 (15.06)	118	0.00
Sport competence change (post–pre)	+0.43 (0.56)	262	+0.45 (0.59)	168	+0.40 (0.50)	94	0.49
Social competence change (post–pre)	+0.18 (0.76)	263	+0.12 (0.78)	170	+0.28 (0.72)	93	0.11
Self-control change (post–pre)	+0.12 (0.83)	265	+0.06 (0.78)	170	+0.22 (0.90)	95	0.12
Effort change (post–pre)	+0.08 (0.70)	267	+0.04 (0.74)	172	+0.16 (0.62)	95	0.17
Teamwork change (post–pre)	+0.07 (0.70)	265	+0.03 (0.69)	170	+0.13 (0.72)	95	0.26
Social responsibility change (post–pre)	+0.20 (0.72)	265	+0.17 (0.76)	170	+0.26 (0.63)	95	0.34
Belonging (post-camp)	4.27 (0.83)	265	4.21	171	4.37 (0.72)	94	0.14
Satisfaction (post-camp)	4.36 (0.89)	263	4.32	169	4.43 (0.86)	94	0.35
Enjoyment (post-camp)	4.38 (0.92)	264	4.34	170	4.47 (0.89)	94	0.26

a *Indicates a p-value based on a univariate X^2^ test, while all other reported p-values are based on univariate t-tests*.

The first binomial logistic regression examined the set of camper characteristics entering camp as predictors of returning status. The characteristics, which are listed in Table 1 included various demographic variables (e.g., age, gender) and levels of key developmental factors entering camp (e.g., pre-camp social competence). Results of the regression showed that the full model (with all predictors included) vs. an intercept only model was statistically significant, χ^2^ (11, *N* = 235) = 23.38, *p* = 0.02. The model correctly classified 88.2% of the non-returners and 28.0% of the returners for an overall correct classification rate of 67.2%. Interestingly, this was only a minor increase relative to the intercept only model which correctly classified 65.1%.

Table 2 shows the logistic regression coefficients (β), Wald test, and odds ratios (including the 95% confidence intervals) for each of the predictors. Only BMI and social responsibility scores at pre-camp were significant predictors in the equation. The odds ratios indicate that, when holding all other variables constant, lower BMI scores and pre-camp social responsibility scores were associated with a greater probability of returning to camp the following year. Overall, these results indicated that the set of variables entering into camp were only marginally good at predicting returning status and that only a few variables–namely BMI and social responsibility–significantly contributed to the prediction.

**Table 2:** Logistic regression results predicting returning status from characteristics entering camp.

**Predictor**	**β**	**Wald *χ^2^***	***p =***	**Odds Ratio**	**95% CI**
Gender (male)	0.04	0.01	0.91	1.04	0.56–1.91
Race (black)	−0.46	1.15	0.28	0.63	0.28–1.46
Poverty status (yes)	0.58	3.00	0.08	1.79	0.93–3.47
Age	−0.08	0.63	0.43	0.92	0.76–1.12
BMI	−0.08	5.86	0.02	0.92	0.87–0.99
Sport competence (pre-camp)	0.10	0.18	0.67	1.10	0.68–1.81
Social competence (pre-camp)	−0.43	2.47	0.12	0.65	0.38–1.11
Self-control (pre-camp)	−0.23	1.40	0.23	0.80	0.54–1.16
Effort (pre-camp)	−0.13	0.20	0.66	0.88	0.51–1.54
Teamwork (pre-camp)	0.03	0.01	0.94	1.03	0.53–1.98
Social responsibility (pre-camp)	0.76	4.61	0.03	2.15	1.07–4.31

*CI, confidence interval*.

The second binomial logistic regression examined the set of variables capturing the campers' participation and experiences throughout the 2018 camp. These variables, which are listed in Table 1, included potential predictors such as attendance, changes from pre- to post-camp in key youth development outcomes targeted in LiFE*sports* (e.g., self-control), and the campers' reflections on camp (e.g., enjoyment of the program). Results of the regression showed that the full model (with all predictors included) vs. an intercept only model was not statistically significant, χ^2^ (10, *N* = 258) = 10.57, *p* = 0.39. The overall classification rate for the intercept only and full model were both 64.0%.

### Qualitative Results

Several themes emerged related to parent/caregiver perceptions of their child's experiences. These themes were categorized into two broader themes initially outlining positive and negative experiences overall. The qualitative analyses for each are described next.

#### Positive Experiences

In relation to positive experiences, five subthemes emerged from the thematic analysis. Each is described here and outlined more specifically in Table 3.

**Table 3:** Qualitative content analysis results.

**Theme and Subtheme**	**Non-returners** **(*n* = 18)**	**Returners** **(*n* = 18)**	**Total** **(*n* = 36)**
**POSITIVE EXPERIENCES**			
**Positive social interactions**	12 (33)	16 (43)	29 (76)
Meeting new people at camp	11 (17)	9 (16)	20 (33)
Improving social skills	3 (3)	8 (12)	11 (15)
Improving communication	3 (3)	2 (5)	5 (8)
Meeting people from different areas of town	3 (4)	6 (7)	9 (11)
Expanding horizons	4 (6)	3 (3)	7 (9)
**Physical activity**	15 (38)	12 (22)	27 (60)
Learning sport skills	6 (8)	6 (8)	12 (16)
Learning new sports	12 (17)	6 (6)	18 (23)
Staying active	7 (10)	6 (8)	13 (18)
Spending time outdoors	2 (3)	0 (0)	2 (3)
**Environmental factors**	9 (16)	6 (8)	15 (24)
Increasing exposure to college	3 (3)	2 (4)	5 (7)
Being in a general positive environment	7 (8)	3 (3)	10 (11)
Being in presence of siblings/cousins	4 (5)	1 (1)	5 (6)
**Logistics**	4 (5)	5 (6)	9 (11)
Benefitting from provided transportation	4 (5)	5 (6)	9 (11)
**Improving eating habits at home**	2 (6)	0 (0)	2 (6)
Healthier eating at home	2 (6)	0 (0)	2 (6)
**NEGATIVE EXPERIENCES**			
**Negative social interactions**	6 (15)	4 (4)	10 (19)
Peer conflict	5 (11)	3 (3)	8 (14)
Alienation	1 (4)	1 (1)	2 (5)
**Logistics**	5 (6)	3 (4)	8 (10)
Difficulty with registration	5 (6)	3 (4)	8 (10)
**Consideration of alternate camps**	5 (9)	7 (8)	12 (17)
Church camps	3 (4)	2 (2)	5 (6)
Recreation center camps	3 (3)	5 (5)	8 (8)
Sport-specific camps	1 (2)	1 (1)	2 (3)

*The first number in each column represents the number of participants who mentioned the theme. The number in parentheses represents the frequency of responses across all participants who mentioned the theme*.

##### Positive Social Interactions

The most common theme discussed by all the parents/caregivers centered around the positive social interactions their children had at LiFE*sports*. This was mentioned by 29 of the 36 participants. The most common theme involved meeting new people (mentioned by 61% of parents/caregivers of non-returners and 50% of returners). A non-returner said, “I think building relationship with peers, learning how to communicate with others and just being in that positive environment is the best part” (N11). Another major subtheme related to positive social interactions focused on improving social skills (mentioned by 16% of non-returners vs. 44% of returners). Other areas mentioned by both groups include improved communication skills, the benefit of meeting youth from other areas, and the benefit of expanding horizons. Responses were fairly similar across the two groups. However, subthemes of improving social skills and meeting youth from different areas of town were more commonly mentioned among parents/caregivers of returners.

##### Sport and Physical Activity

Another major positive theme focused on physical activity involvement. Most commonly, interviewees noted themes of learning new sport skills, learning new and different sports, and staying active. Specifically, 6 of each of the 18 non-returners and returners mentioned the benefit of youth learning new sport skills, which represented 33% from each group. More commonly, 66% of non-returners noted the importance of learning new sports. A non-returner said, “Engaging in different sports. He's eager to do different ones rather than sticking to just that one. He's open so I think he's being active and learning how to play different sports. I think that was the main skill” (N9). Only 33% of returners mentioned this benefit, as one stated, “Well she got a little bit of knowledge of different sports that she wouldn't normally play like lacrosse” (R9). Additionally, both groups recognized youth staying active throughout LiFE*sports* summer camp with 38% of non-returners and 33% of returners noting its importance. A non-returner said, “The best part is that he is constantly being active for a number of hours throughout the day and knowing that it was a positive,” (N9) and a returner shared, “Well he is a big fan of sports so mainly because of the sports and it keeps him active during the summer” (R8). Two (11%) non-returners also spoke on the benefit of spending time outdoors yet it was not mentioned by any parents/caregivers of returners.

##### Environmental Factors

Several parents/caregivers in both groups spoke favorably about environmental aspects of the program. The themes that emerged were the increased exposure to college that LiFE*sports* offers, the general positive environment, and the presence of siblings and cousins at camp. Of the parents/caregivers of non-returners, 16% noted the benefit of exposure to college. One non-returner said, “I like that he got exposure to OSU campus because I'm trying to convince him that that's where he wants to go” (N14). Similarly, 11% of returners noted this benefit. More non-returners spoke of the general positive environment as a benefit to the youth as 7 of the 18 (38%) of the non-returners mentioned it and only 3 of the 18 (16%) of returners. A non-returner shared, “I like that he is going somewhere, and everyone is positive” (N9) and a returner said, “I decided to send them back because of the positive experience that they had. I also went when I was younger, and I has a good experience” (R3). Lastly, the presence of siblings and cousins was mentioned as a benefit to the environment. Far more non-returners mentioned this subtheme, at 22%, whereas only 5% of returners spoke of it.

##### Logistics

The ease of transportation was noted as a benefit mentioned by 22% of non-returners and 25% of returners. Both groups valued the transportation provided and highlighted it as a positive aspect of the LiFE*sports* camp.

##### Healthier Eating Habits at Home

A total of 2 non-returners spoke of the impact that attending LiFE*sports* summer camp had on healthy eating habits at home. For instance, an individual mentioned, “She learned stuff about that and eating healthy and came home and was telling me how to eat better” (N5). This topic was not mentioned by any returning interviewees.

#### Negative Experiences and Challenges

In relation to negative experiences and/or challenges, three major themes emerged from the content analysis. Each is described here and outlined more specifically in Table 3. Additionally emergent themes from the follow-up question to parents/caregivers of non-returners related to external circumstances also are described.

##### Negative Social Interactions

A total of 10 interviewees (27%) spoke on negative social interactions that took place at the program, including 6 parents/caregivers of non-returners and 4 of returners. A few differences between groups of interviewees were noted. Five non-returners (27%) mentioned how their child experienced peer conflicts at camp as compared to only 3 returners (16%). Four of the non-returners mentioned few situations where their child was directly victimized, whereas two highlighted generic issues (i.e., other youth were unruly or rude). A non-returner said “My daughter had a bad experience with pushing and not feeling safe. It was brushed over when I called to discuss it so I didn't feel good sending my kids back” (N17). Of the three parents/caregivers of returners that mentioned issues with peer conflict, two noted that their child was a direct victim of an issue. The other returner mentioned generic issues of other youth fighting but said this did not directly impact their child. As an example, one parent/caregiver of a returner said, “I think maybe there was a time when my son had issues with one kid but it was resolved that day” (R17). Additionally, two parents/caregivers (one of a non-returner and one of a returner) mentioned examples of alienation.

##### Logistics

Several interviewees noted logistical challenges hindering their child's return to summer camp. Eight of the 36 (22%) noted difficulty with the registration process, including 5 non-returners and 3 returners. A non-returner said, “We forgot the date, too late to register in the afternoon by the time my wife realized it was on that day. Because they take a certain number of kids, so it was too late” (N16). Fewer returners noted issues with registration, yet one said, “Just with getting him signed up I guess because it is first come first serve, so I have to make sure that he's all squared away” (R8).

##### Consideration of Alternate Camps

Both parents/caregivers of non-returners (27%) and returners (38%) reported on other camps their child had alternatively considered that summer. Several subthemes emerged, focusing on alternative camps through churches, recreation centers, and sport-specific camps. A parent/caregiver of a non-returner shared, “He went to a church camp, but it wasn't an all-day camp had to pick him at 1 pm but it was closer to home” (N2). One parent/caregiver of a returner mentioned, “Yes we looked at the YMCA camp and the Columbus Parks and Recreation, but she wanted to come back to LiFE*sports*” (R9).

##### External Circumstances

Parents/caregivers of non-returners were asked a follow-up question related to whether there were any external circumstances that prevented their child from returning to the program. Of the 18 non-returners, 16 explicitly mentioned a circumstantial reason as to why their child did not come back to camp (ranging from missing the registration date to spending the summer with family out of state). The most commonly reported theme involved family schedule conflicts (mentioned by 8 non-returners). One stated, “Just a busy summer and lots of vacations so we didn't have time for her to go back to camp” (N13). Four interviewees state they intended for their child to return but missed the day of registration making them ineligible to attend. As one reported, “We forgot the date, too late to register in the afternoon by the time my wife realized it was on that day. Because they take a certain number of kids, so it was too late” (N16). Two parents/caregivers of non-returners mentioned negative interactions with peers impacted their child's re-enrollment. She stated, “My daughter had a bad experience with pushing and not feeling safe. It was brushed over when I called to discuss it so I didn't feel good sending my kids back” (N17). Nine interviewees had alternative plans for their child, such as attending a sport skill-specific camp, spending time with family out of the area, or attending summer school. These alternative plans were not made as a result of not desiring to return to camp but still prevented the youth from returning. These external factors that prevented the youth from returning personify the difficultly in predicting retention in vulnerable populations.

## Discussion

Previous studies have found that sport-based PYD programs yield many positive outcomes for socially vulnerable youth, including improved fundamental motor skills, perceived physical literacy, physical competence, physical self-worth, social-emotional learning skills, and prosocial behaviors (e.g., Eime et al., 2013; Anderson-Butcher et al., 2014b, 2018; Hermens et al., 2017; Whitley et al., 2019; Warner et al., 2021). Long-term engagement is one key to youth benefiting from sport-based PYD programs (Anderson-Butcher, 2005). Although retention is important in promoting positive outcomes such as increased physical literacy, little research has been conducted on retention in sport-based PYD programs, as well as other programs designed to promote physical literacy and related outcomes. This study, therefore, addressed this gap in the literature by exploring factors contributing to retention among socially vulnerable youth attending one sport-based PYD program.

### Overall Findings

When exploring differences in characteristics among returners and non-returners, difference tests indicated non-returners were less likely to be Black and more likely to live in poverty. Non-returners also were slightly older, had higher BMIs, and reported lower perceived sports competence pre-camp than returners. This relates to previous research that has identified poverty and higher BMI as risk factors for retention and positive developmental outcomes (Ullrich-French and McDonough, 2013). Certainly, age has long been associated with dropout (Anderson-Butcher and Fink, 2005) and poor competence can be a barrier to continued participation (Anderson-Butcher et al., 2006). Effect sizes in the present study, however, were small (ranging from 0.01 to 0.04), suggesting these differences between returners and non-returners may have little practical significance.

In relation to experience and participation, difference tests found support for overall attendance at the previous year's camp, as returners attended more often the previous year than non-returners (again with a small effect size). Perhaps youth with greater attendance levels had more positive interactions at the camp. Indeed, continued participation allows for more opportunities for positive, as well as negative, experiences, which may impact retention, and would be consistent with prior research (Hansen and Larson, 2007). The value of change occurring, as well as likelihood of continued participation, has emerged in other research. For instance, Coalter's qualitative research points to program theories emphasizing the value of social relationships for promoting attitudinal, value, and behavior change (Coalter, 2012). Anderson-Butcher and colleagues (Anderson-Butcher et al., 2003, 2014b, 2020; Anderson-Butcher and Fink, 2005) continues to demonstrate relationships, including those foster a sense of belonging, are central to promoting attendance and mediating the relationship between participation and outcomes. Implications for the design of sport-based PYD programs inclusive of strategies to support social interactions and positive relationships are clear.

Additionally, regression analyses found better fitness levels and more favorable pre-camp perceptions of social responsibility were associated with greater probability of returning. This suggest youth coming into the program with skills targeted by the program may be more likely to continue in their involvement, supporting Hellison and Martinek's (2009) concept of cultural matches. Although findings point to some significant factors, the overall effect sizes across all analyses were small. As such, findings suggest these variables have limited importance in relation to explaining whether campers return in subsequent years. However, certainly there is evidence that youth “vote with their feet” and may leave sport settings as interest wanes yet be drawn to other social settings such as work or the arts that also may promote positive outcomes (see Weiss and Ferrer-Caja, 2002; Eccles et al., 2003; Crane and Temple, 2015).

Interviews with parents/caregivers of returners and non-returners provided some insights in relation to the experiences at the prior year's camp among the youth. Specifically, parents/caregivers of returners and non-returners reported their children had positive experiences at LiFE*sports* at fairly similar rates. Their reporting of prosocial interactions (such as meeting new people which was mentioned by over 2/3 of all parents/caregivers) was positive overall. When further examining the frequency of comments, parents/caregivers of returners were more likely to mention social skills improvements among their children and the value of meeting people from different areas of town. However, this contradicted the quantitative results which did not identify any of the social skills targeted at LiFE*sports* as factors related to retention. More research is needed to understand how growth social skills and other desired program outcomes may or may not contribute to retention. However, findings here further point to the value of social interactions and interpersonal relationships as a critical component of sport-based PYD program design.

Both sets of parents/caregivers also positively spoke about the program's physical activity and sport components, reporting their child learned new sports and sport skills at LiFE*sports*. Indeed these parents/caregivers reported on the value of LiFE*sports* for promoting physical literacy. Interestingly those of non-returners mentioned these outcomes more often than those of returners. Quantitative analyses support the importance of perceived sport skills for predicting whether youth came back in 2019 (although effect sizes were small), but also did not find relevance for changes in perceived athletic competence over the course of participation. However, qualitative results found parents/caregivers of non-returners mentioned the value of physical literacy outcomes (as well as healthy eating behaviors) more often. Findings related to physical literacy's role in retention are still unclear, and more research is needed to understand how unique sports and the learning of techniques and tactics can be leveraged to improve retention. One wonders, however, what moderators are important to explore in future research and practice. Many (Eccles et al., 2003; Anderson-Butcher et al., 2020) have called for a better understanding of what programs work for whom and under what conditions. Certainly individualizing programming to meet the needs (as well as interests) of the targeted population of youth being served continues to be a priority for practice.

In building from these findings, one translational piece on recruitment and retention in PYD programs provides further insights for practice. Using self-determination theory, achievement goal theory, and competence motivation theory (Harter, 1978; Ryan and Deci, 2000; Treasure, 2001), Anderson-Butcher (2005) describes five key factors important for youth motivation in PYD, including the presence of mere opportunities; interest/relevance of the achievement context, opportunities to develop and demonstrate competence, experiences of autonomy-support, and relationships. The author concludes by suggesting that programs use diversified straegies to attract a variety of young people, find individualized matches between youths' needs, interests, and the program design, and realize “one size fits all” approaches will not work. Creativity in programming and program retention strategies are needed, especially to engage the hardest-to-reach youth.

Parents/caregivers of returners and non-returned reported negative experiences and challenges at similar rates. About a third of the parents/caregivers in each group also described how the presence of other summer camps offered at the same time as LiFE*sports* was a challenge. Parents/caregivers in each group mentioned difficulties with program registration at similar rates. The only major difference, albeit small, related to negative social interactions. Specifically, parents/caregivers of non-returning youth mentioned peer conflict more frequently than those of returning campers. This corroborates previous research that has found that peer conflict and poor team dynamics increase dropout rates in sport (Fredricks et al., 2010; Crane and Temple, 2015). Additional questions asked of parents/caregivers of non-returners suggested they may be more likely to have family schedule conflicts or miss registration day. Competition with other camps and summer programs continues to be a barrier to participation, as found in other research (Crane and Temple, 2015).

In the end and similar to the quantitative findings, the thematic analyses did not really differentiate experiences between returners and non-returners. Positive and negative experiences, in general, were reported at similar rates by the parents/caregivers of both groups. In most cases, comments from those of non-returners were more favorable. Logistical issues within the program seem to be important to address, such as transportation and the registration processes. Also, social interactions seem to be an important part of the program, and conflicts with peers and others do seem to be a deterrent to involvement. Future research should address these barriers and explore how improved social relations and peer/team dynamics impact outcomes and ultimately choices to continue participation. Understanding the realities of families living in poverty who are exposed to other social vulnerabilities continues to be a priority for sport-based PYD research and practice.

### Limitations and Next Steps

While our study did not show much support for the factors studied here in relation to retention, no doubt there are other variables not explored that might influence parents/caregiver and children's decisions to continue participation. A better understanding of the role of peers and social interactions and their impact on retention certainly seems a worthy next step for research. Additionally, the context of this study also may be a limitation. Only one sport-based PYD program was examined, and this program serves mostly urban Black youth living in poverty. Perhaps the anomalies of predicting retention in this context and with this group of young people are more challenging than would be in other settings and with other subpopulations of youth.

There also may be other issues limiting the validity of study. For the quantitative study components, data were gathered using youth self-report measures. While self-report measures are important for capturing youth perceptions and have been supported in the literature (e.g., Paulhus and Vazire, 2007), they are susceptible to response bias, social desirability bias, mono-method bias, and systematic bias which can cause measurement error (Chan, 2009). Additionally, the youth may have experienced response biases given the large number of items on the survey. Perhaps these measurement issues brought additional error to the analyses which future research should consider.

Further, sampling was definitely an issue with the qualitative portion of the study. Challenges persisted with parent/caregiver recruitment, and perhaps the ones who agreed represented families with children who had more favorable experiences in the program. Narratives of those who were less satisfied with the program may not have been captured. Additionally, future research may wish to consider the voices of youth through qualitative research, thereby better understanding their motivations for returning. From a big picture perspective, we also acknowledge that sport-based PYD programs, such as the one studied here, are typically limited in duration. As such, it is unclear whether these program will result in long-term impact on outcomes such as physical health in adulthood or lifelong physical activity participation. This should be an area of future research. Similarly, research has found effective, well-evaluated evidence-based PYD programs, in general, are most effective when delivered over a period of 9 months or more (Catalano et al., 2004). However, this same review found how interventions shorter than 9 months were most effective when averaging 12 sessions in total (much less than the 19 sessions of sport-based PYD program studied here).

## Conclusion

In conclusion, study findings demonstrate the challenges associated with retaining young people's involvement in sport-based PYD and other related programs designed to address physical literacy. Even our exploratory mixed methods study had difficulty finding predictors and meaningful experiences that differentiated returners and non-returners. Better fitness levels, higher perceived social responsibility, and some demographic variables (such as lower poverty rates and younger age) were associated with a greater probability of returning, although effect sizes were small. Parents/caregivers of returners and non-returners reported similar rates of positive and negative experiences with the programs, with only small differences found in relation to social interactions (with returners reporting more favorable ones) and the value of the physical literacy components (with the non-returners reporting more favorably). Findings overall demonstrate challenges with predicting retention and fostering long-term engagement among youth from socially vulnerable circumstances in programming. Typical program qualities and evidence-based practices in sport-based PYD programming (i.e., belonging, satisfaction, etc.) may not factor into decisions about returning for youth coming from diverse, socially vulnerable backgrounds. Perhaps the stressors and challenges faced by families struggling with poverty and its correlates may prevent even well-designed retention efforts from working.

## Data Availability Statement

The raw data supporting the conclusions of this article will be made available by the authors, without undue reservation.

## Ethics Statement

The studies involving human participants were reviewed and approved by the Ohio State University Behavioral and Social Sciences Institutional Review Board. Written informed consent to participate in this study was provided by the participants' legal guardian/next of kin.

## Author Contributions

DA-B, AA, and TS: conception or design of the work. CS, EG, DA-B, AA, and TS: data collection, data analysis, and interpretation. DA-B, AA, TS, CS, and OA: drafting the article. DA-B: critical revision of the article. All authors contributed to the article and approved the submitted version.

## Conflict of Interest

The authors declare that the research was conducted in the absence of any commercial or financial relationships that could be construed as a potential conflict of interest. The handling editor CQ-Y declared a shared affiliation with several of the authors DA-B, CS, TS, OA, and EG at time of review.

## Publisher's Note

All claims expressed in this article are solely those of the authors and do not necessarily represent those of their affiliated organizations, or those of the publisher, the editors and the reviewers. Any product that may be evaluated in this article, or claim that may be made by its manufacturer, is not guaranteed or endorsed by the publisher.
