# Supplementary material for: What Keeps Kids Coming Back? Retention in a Sport-Based Positive Youth Development Program
Source: Front Sports Act Living. 2022 Jul 20;4:816539. doi: 10.3389/fspor.2022.816539 (PMC9347661; doi:10.3389/fspor.2022.816539)
Supplement: Supplementary file 1 [file Data_Sheet_1.PDF]

CHALK TALK

lessons to enhance youth social skills

LiFEsports™
at The Ohio State University®

SESSION 5: REGULATING & CONTROLLING YOUR EMOTIONS

Overview

Today's session is about regulating and controlling emotions. By teaching the youth how to control their internal voice or self-talk, they can begin to practice self-control in everyday life. The ability to practice self-control will help create better self-awareness, build positive social interactions, and contribute to overall health.

Helpful Hints for the Chalk Talk Coach

During last session, youth identified ways in which their body reacts to emotions. Pay particular attention to group members who shared extreme body reactions because these people likely engage in more negative self-talk. As you work through the activities, be sure to help youth identify the truth and lies in their own self-talk. Explain how the "voices" in our heads often exaggerate.

Intended Outcomes

- Youth will understand how internal thoughts (self-talk) affect emotions.
- Youth will be able to identify positive and negative self-talk.
- Youth will be able to identify strategies to help them regulate their emotions.

Equipment

- Two liter of soda (9 to 10 year olds only)
- Two liter of water (9 to 10 year olds only)
- 2 packs of Mentos (9 to 10 year olds only)
- 8 Mantra Posters (11 to 12 year olds only)
- Business card-sized pieces of paper (11 to 12 year olds only)
- Playbook (All ages)

OUTLINE OF SESSION

Review of Previous Sessions | 5 minutes

Take a few minutes to review what the youth have learned so far in LiFE*sports*, such as rules and expectations, non-verbal cues, and different types of emotions. Follow up on last session's daily challenge: ask the group members to share examples of times during LiFE*sports* or at home when they felt emotions and their bodies had a physical reactions.

Remind the group that today will be the last lesson on self-control. Explain that today's session will build on the previous two sessions about self-control because youth will learn how to control or regulate their emotions and their body's responses to those emotions.

Knowledge | 5 minutes

Explain to the group that to fully gain control of emotions, it is important to first name the emotion that we feel and then understand how our own beliefs and self-talk play into our response to that emotion. If we understand how our thoughts (spoken and unspoken) shape our strong emotions, we can regain control of our emotions and figure out the best way to respond.

What is Self-Talk?

Ask the youth: what do you think self-talk is? Then share an example of your own use of self-talk and ask for examples from the group members.

Definition: Self-talk is the act of speaking to one's self out loud, silently, or mentally (in one's head)

Example situation: "I am feeling worried about a big math test."

Negative self-talk example: "I am going to fail, I can't do this, I didn't study, I will never pass this test."

Positive self-talk example: "It is just a test, I can do this, relax, chill, I got this, I will try my best."

AFTER SHARING EXAMPLES, ASK THE FOLLOWING QUESTIONS ABOUT THE TWO TYPES OF SELF-TALK:

- ★ How do these make you feel?
- ★ Do you feel better when speaking positive?
- ★ Do you feel calmer?

Describe how positive self-talk allows us to collect our thoughts, recognize our strong emotion, and get back in control. If we use negative self-talk, it is more likely that our emotions will get out of control.

SHARE THE FOLLOWING ACRONYM AND EQUATION WITH THE GROUP:

A — ACTIVATING EVENT *(Example: My friend asks someone else to be his partner in a game)*

\+

B — BELIEF *(Example: No one likes me, I have no friends)*

=

C — CONSEQUENCE *(Example: Feeling depressed, sad, hopeless)*

Ask the group: what would happen if we changed the belief or the self-talk in the equation? How might the consequence change? Explain that today's activities are intended to help them identify their own beliefs and self-talk so they can develop strategies to make their self-talk positive.

ACTIVITY 1 | 20 minutes

BOTTLED UP EMOTIONS FOR 9 TO 10 YEAR OLDS

Give each group member two slips of paper: one to write a positive self-talk statement and one to write a negative self-talk statement. Ask the group to label each as + (positive) or – (negative). Then, tape the negative statements to a 2-liter bottle of soda. Add a few Mentos to the bottle of soda. Explain that this reaction is similar to what happens to our minds and bodies when we use negative self-talk. Tape the positive statements to the 2-liter bottle of water. Add a few Mentos to the bottle of water and explain that the reaction is similar to what happens to our minds and bodies when we use positive self-talk.

MY MANTRA FOR 11 TO 12 YEAR OLDS

Prior to this activity, make eight large poster boards with mantras written on them to mark eight stations. To get started, divide the group members among the eight stations. Explain that a mantra is a statement that helps define who or what something is and this activity helps us learn about how mantras can help us with positive self-talk. Give the group seven minutes to go around to all of the stations and guess who or what is associated with each mantra. Also, have them write a situation on the poster board when they could use that mantra. Below are a few examples:

1. Just Do it (Nike)
2. Be All That You Can Be (The US Army)
3. Play Like A Champion Today (Notre Dame Football)
4. Strive For Greatness (Lebron James)
5. There is No Try, Only Do (Yoda)
6. Let it Go (Frozen)
7. Take a Deep Breath and Walk (Taylor Swift)
8. Every Journey Begins with a Single Step (Maya Angelou)

After they have visited each station, reveal the authors of the mantras and review some of the group's examples. Allow them to share first and then read a few examples from each poster board. Make sure to emphasize how positive self-talk (like these mantras) helps us calm down, remain positive, and regain control of our emotions. Then, have the youth pick a favorite positive self-talk phrase and write it down on a business card- sized piece of paper. They can spend time decorating their favorite phrase for the last few minutes of the activity.

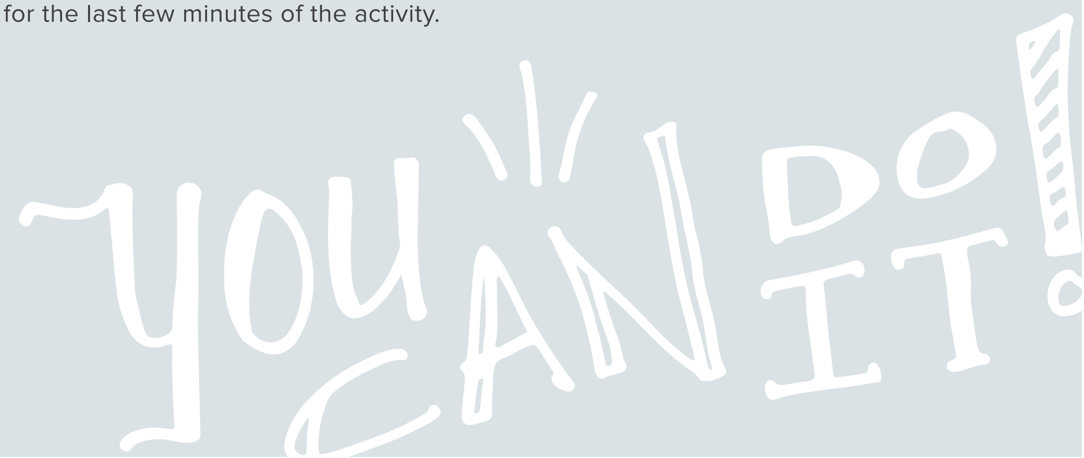

ROLE PLAY FOR 13 TO 14 YEAR OLDS

Divide the group into four groups. Give each group one of the following scenarios:

- You miss the game winning shot in your basketball game.
- You win the most improved award on your soccer team.
- You fail a test at school.
- You win the 400 meter race in your track meet.

Instruct the youth develop a two to three minute skit that includes examples of both positive and negative self-talk that could occur in each of these scenarios. Give them 10-15 minutes to develop the skit, and then ask each group to act out its skit. After all of the groups have presented their skits, reflect on which type of self-talk they might have in each situation and how they can train themselves to use the positive self-talk messages.

Conclusion and Group Processing | 5 minutes

Take a few minutes to discuss the activities. Ask the following key processing questions:

- **WHAT:** When was one time you had to control your emotions?
- **SO WHAT:** Were you able to control your emotions? Why or why not? Did you have positive or negative self-talk?
- **NOW WHAT:** How can you take the strategies you learned today to control or regulate your emotions and use them at school or at home?

Summarize the day by reviewing the concept of ***Activating Event + Belief = Consequence.*** *Ask the group to come up with a few more examples, if time permits.*

Self-Reflection | 15 minutes

At the end of each social skill unit, we ask the youth to journal about his or her time in Chalk Talk. This is done using the playbook. Journaling will give the youth time to reflect on what they learned through the activities and how the skills they've learned relate to other situations in their lives. Please give one playbook to each youth and instruct him or her to spend 10-15 minutes writing about what he or she learned about self-control.

Ask them to respond to the following prompts:

- What does it mean to have self-control / what does self-control look like?
- Think of a time when in sports, at home, or at school when you showed self-control. Describe how you knew you were showing self-control. What happened when you showed self-control?

★ What would have happened if they didn't?

Also, let them know that they are free to draw in their playbook if they do not feel like writing.

Additionally, this is the end of the self-control unit. Remind youth that this unit involved three sessions focused on self-control, the first letter in ***SETS***. They should begin to find ways to apply ***SETS*** at home.

HIGHLIGHT LESSONS LEARNED AND KEY IDEAS FROM:

SESSION 1	SESSION 2	SESSION 3	SESSION 4
Introduction to LiFE*sports*	Overview of SETS & Establishing a Team Culture	Recognizing Emotions	Understanding Your Body's Reaction to Emotions

You can also go over group norms, review names, and practice skills like the calming techniques.

★ **DAILY CHALLENGE** ★

Identify one time during Chalk Talk or at home that you heard yourself use positive or negative self-talk.

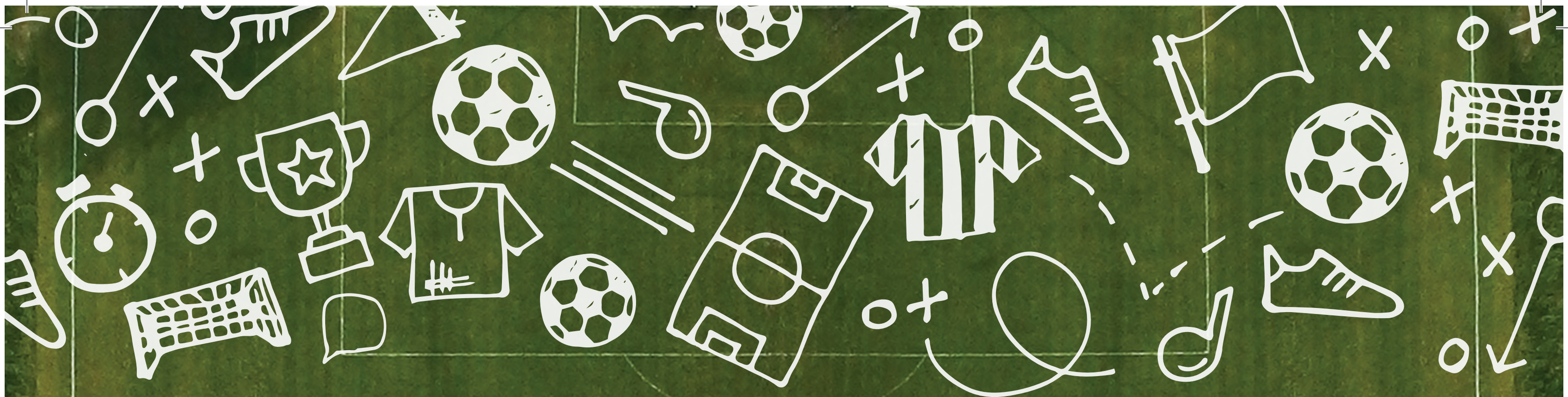

SOCCER LESSONS

lessons to enhance youth sport and social skills

LiFEsports™
at The Ohio State University®

SESSION 6: DRIBBLING & BALL SKILLS

Overview
Today's lesson will be the first session where you will repeat skills taught previously. You will work with youth today on dribbling and ball skills. Youth should focus on increasing their ability to keep the ball under control when dribbling. You should actively engage youth in discussions on if they are seeing any improvement in their performance or ability. Today's soccer activities also provide the opportunity to demonstrate the social skill of effort to youth, especially the relay races.

Sport Skill Outcomes

- Youth will understand the importance of balls skills and dribbling in soccer.
- Youth will be able to dribble the ball with their eyes looking forward.
- Youth will be able to maintain control of the ball and keep it close to their body while in motion.
- Youth will be able to describe the cues for dribbling.

Equipment

- 15-20 cones
- One ball per youth
- Pinnies

OUTLINE OF SESSION

Framing | 5 minutes
Explain to the group that today's focus is on practicing dribbling and ball handling skills. If you are using these lessons in an afterschool program, use this time to also reflect on the games during the previous lesson.

Sport Skill Overview & Demonstration | 10 minutes
Begin by demonstrating to the youth how to dribble using the cues below. You can chose a youth to demonstrate if you have a child you has played soccer before.

Dribbling & Ball Skills

- Push the ball with the inside of the foot.
- Keep your eyes looking forward, not down at the ball.
- Keep the ball close ("under your nose"), within arm's length of the body unless dribbling with speed.
- Use both feet when dribbling.

Once you have completed the demonstration, ask the group if there are any questions. If there are no questions, or when all questions have been answered, begin the activities.

ACTIVITY 1 | *15 minutes*

TEAM RELAYS

Prior to beginning the activity, set up five lines of ten cones each with the cones about two feet apart. Divide the group into five smaller groups. Each person will take their ball and dribble in and out of the cones using the inside and outside of their foot in an alternating motion or using both the left and the right foot. Make sure to emphasize proper dribbling technique.

Once the youth have practiced going through the cones a few times each, then turn the activity into a relay race. Have each team go through the cones (one at a time) as fast as they can. When each team member gets back to the start have him or her sit down so you can tell which team finishes first. Run through the relay activity a few times and mix it up, such as using only the right foot, only the left foot, etc.

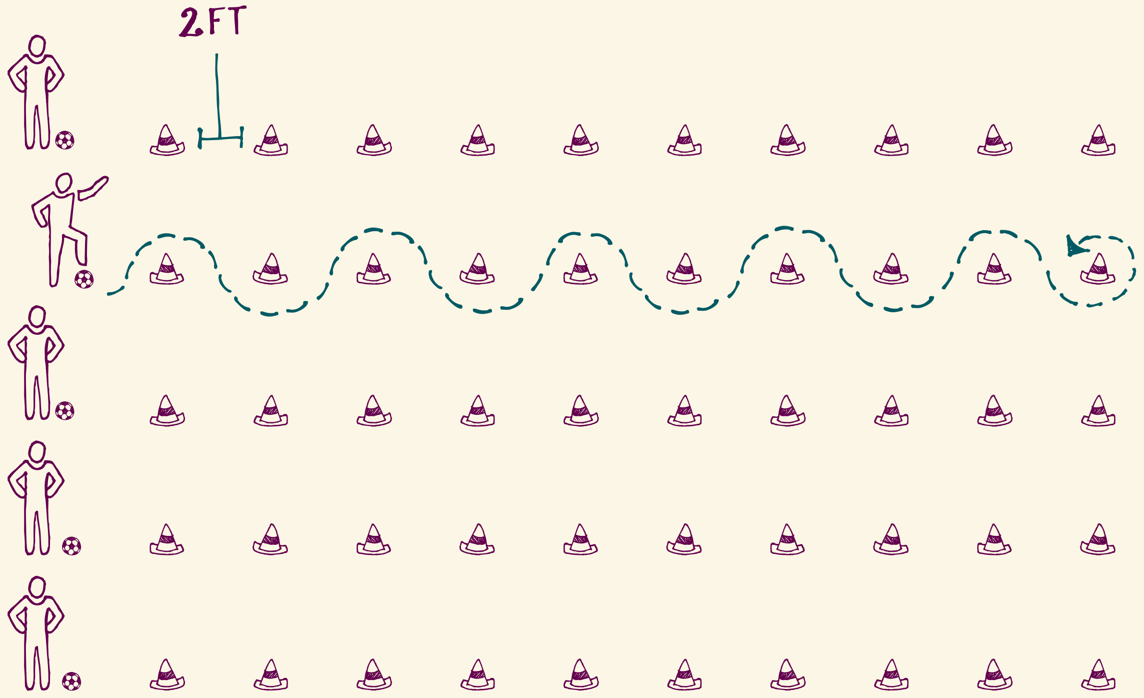

Helpful **HINT!**

Since this will be the second time the youth have concentrated on dribbling, focus on their continued growth and development of this skill. Make sure they are practicing the skill correctly and provide feedback to correct their skill as needed. For instance, you might say, "Good job using the inside of foot, but remember to keep your eyes up to look where you are going." Also, continue to emphasize when they demonstrate any of the *SETS* because as they develop their sport skills, they understanding of how *SETS* applies will increase as well.

ACTIVITY 2 | 20 minutes

PAC MAN

Prior to the activity, create four squares with four cones each 10 yards apart. Each square should be wide enough that four youth can be inside at once with enough space to dribble and control the ball, but not too wide to make sure the drill is challenging. Divide the group into two equal teams and assign four people to be the defenders. Each of the youth will have their own ball (except the defenders). Ask the four defenders to place themselves in each square of cones.

Instruct the youth with soccer balls to continually dribble in a clockwise motion through the defenders' squares. The object of the game is to keep control of the ball while avoiding the defenders. Each square they get through successfully earns them one point. They do not get any points if they lose control of the ball or if it goes out of bounds. After a few minutes, rotate the players so there are new people playing defense. Emphasize correct dribbling cues during this drill.

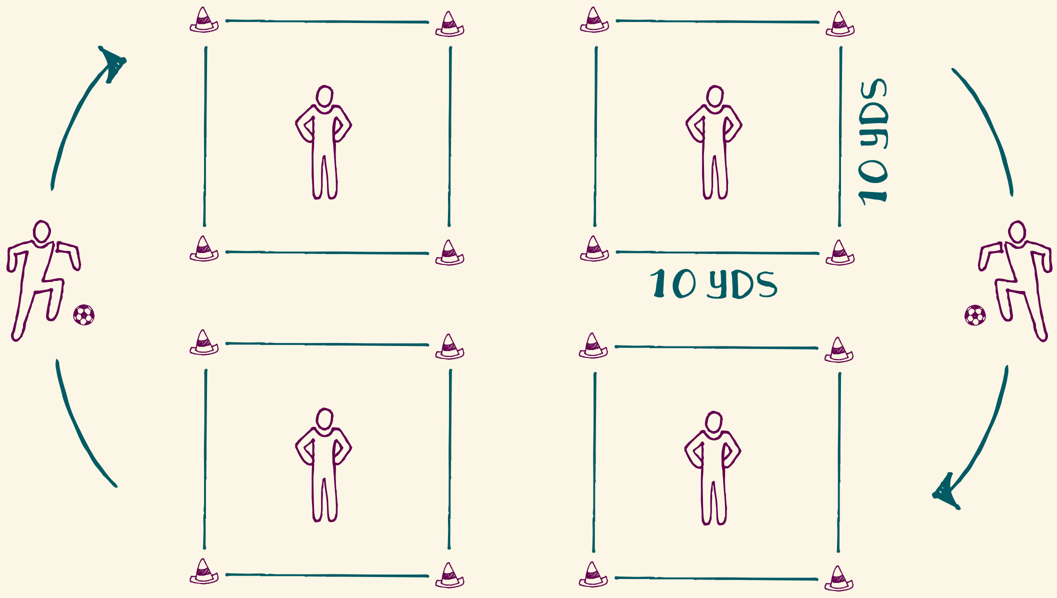

Debriefing | 10 minutes

Take a few minutes to process today's activities. Make sure to connect today's activities to the social skill of self-motivation as it relates to effort. Key process questions include:

- **WHAT:** What were the cues for dribbling and ball skills? What ***SETS*** were used during the activities?
- **SO WHAT:** Why was it important to be self-motivated when you were dribbling and practicing ball skills? How did you have to use effort to be successful? When you worked hard, did it make a difference?
- **NOW WHAT:** Why do you think it is important to have self-motivation when you are working on school homework?

Once you have processed these questions with the team, make sure to preview the next lesson's sport skill (passing and receiving the soccer ball).
